# Effects of perioperative hydrogen inhalation on brain edema and prognosis in patients with glioma

**DOI:** 10.3389/fneur.2025.1625636

**Published:** 2025-07-09

**Authors:** Liu Dong-mei, Yao Jian-qiang, Ma Yan-e

**Affiliations:** Yulin City First Hospital, Second Affiliated Hospital of Yanan University, Yulin, Shaanxi, China

**Keywords:** hydrogen inhalation, glioma patients, brain edema reduction, neuro-oncology, perioperative care

## Introduction

Wu et al. (1) conducted a pioneering randomized controlled trial (RCT) to evaluate perioperative hydrogen inhalation in glioma patients, demonstrating a statistically significant reduction in postoperative brain edema and improved short-term neurological outcomes. While these findings highlight hydrogen's potential as an adjuvant therapy, critical analysis from statistical and clinical perspectives is necessary to assess the validity of conclusions and guide future research. This commentary addresses methodological limitations, contextualizes the results within existing evidence on hydrogen therapy, and proposes directions for advancing this field.

## Discussion

Strengths and statistical considerations: the study's RCT design and focus on objective outcomes such as edema volume via MRI strengthen its reliability. However, several statistical limitations should be considered for attention: sample size and generalizability: the single-center trial enrolled 120 participants, which is modest for evaluating clinical outcomes in heterogeneous glioma populations. Larger multicenter studies in glioma populations, such as those in Parkinson's disease (2), are essential to validate these findings across diverse demographics and tumor subtypes; Blinding and bias control: Although randomization was implemented, the lack of detailed blinding protocols such as whether outcome assessors were blinded raises concerns about measurement bias, a common issue in hydrogen therapy trials (3). The comprehensive and explicit documentation of allocation concealment along with blinding methodologies is of particularly importance for ensuring the replicability of research findings; Clinical vs. Statistical Significance: while a 15% reduction in edema volume was statistically significant (*p* < 0.05), its clinical impact remains unclear. Future studies should prioritize patient-centered outcomes, such as functional recovery or survival rates, to align with real-world therapeutic goals (4). Mechanistic and clinical context: hydrogen's antioxidant and anti-inflammatory properties, well-documented in preclinical studies (5), provided a reasonable mechanism for reducing edema. While Wu et al. (1) reported reductions in oxidative/inflammatory markers (MDA, SOD, IL-6, TNF-α), the absence of mediation analysis precludes definitive causal attribution of edema reduction to these mechanisms. Integrating such biomarkers, as seen in trials of hydrogen water for neurodegenerative diseases (2), would enhance mechanistic clarity. Clinically, the study's short follow-up (30 days post-surgery) limits insights into long-term prognosis. Glioma management prioritizes survival and quality of life; Thus, extending observation periods to 6–12 months is critical to evaluate hydrogen's durability. Additionally, comparing inhalation with other delivery methods such as oral hydrogen-rich water could optimize therapeutic strategies. For instance, inhalation may offer rapid bioavailability during surgery, while oral administration might suit long-term home use (6). Translational challenges and future directions: the authors appropriately highlight hydrogen's safety profile, but scalability challenges still exist. Hydrogen inhalation requires specialized equipment and monitoring, increasing healthcare costs compared to oral alternatives. Cost-effectiveness analyses are needed to justify clinical adoption, especially in resource-limited settings. Future research should focus on: dose optimization: the study used a fixed hydrogen concentration (67% H_2_/33% O_2_ mixture). Exploring dose-response relationships could maximize efficacy while minimizing risks; Combination therapies: pairing hydrogen with standard treatments such as temozolomide may synergistically enhance outcomes, as suggested by preclinical models (5); Patient stratification: subgroup analyses based on glioma molecular subtypes such as IDH mutation status may identify populations most likely to benefit.

## Conclusion

Wu et al. (1) provided valuable preliminary evidence supporting hydrogen's role in glioma care. However, methodological rigor, mechanistic validation, and translational feasibility require further exploration. By addressing these gaps, hydrogen therapy could evolve from an experimental intervention to a clinically impactful adjuvant in neuro-oncology.
